# “Here I can just be myself”: How youth and adults collaboratively develop an identity‐safe community across difference

**DOI:** 10.1002/jcop.22526

**Published:** 2021-02-24

**Authors:** Jennifer D. Rubin, Mike Scanlon, Anna Cechony, Katharine Chen

**Affiliations:** ^1^ Foundry10 Seattle Washington USA

**Keywords:** community building, identity safety, positive risk‐taking, underrepresented youth, youth–adult partnerships

## Abstract

The aim of this study was to investigate the development of identity safety—where all participants are valued, included, and can engage without fear of stigmatization—among underrepresented youth and adults in a community‐based youth development program. Qualitative in‐depth interviews were conducted daily with three youth and two adult mentors about their experiences in the program (a total of 32 interviews). Data analysis revealed that participants developed identity safety through engaging in programmatic activities that explored youth's identities, practicing authenticity in daily interactions, and facilitating dynamic communication across intergenerational friendships. Participants described sustaining identity safety through formal social support spaces, mutual support in group settings, and peer support. Ultimately, these experiences set the foundation for youth and adults to engage in positive risk‐taking and self‐reflection. Implications for researchers and youth development programs are discussed.

## 1 INTRODUCTION

Community‐based organizations often take measures to create inclusive environments, especially for young people belonging to underrepresented groups. These efforts range from designated programming for underrepresented youth to mentorship experiences that celebrate multicultural values. Yet, a remaining challenge in creating inclusive environments is ensuring these settings function so that youth in them feel safe and connected. For youth whose identities have been historically invalidated, these community settings can prompt a basic question: How will this community affirm one's lived experience?^1^

We situate our research in an assumption about social contexts: community settings can signal the degree of safety that a young person will experience. That is, certain cues in community settings can either affirm or devalue youths' lived experiences and social identities (Davies et al., 2005; Steele & Cohn‐Vargas, 2013). A substantial body of research has found that identity‐safe contexts—settings that acknowledge differences in social identity and treat those differences as valuable—are an important component for development among underrepresented youth (Gamarel et al., 2014; Lowe, 2020; Purdie‐Vaughns et al., 2008). However, less is known about how diverse groups of youth and adults *collaboratively develop* identity safety in a community setting.

In the present research, we explore the ways in which a diverse group of youth and adults in the U.S. collaboratively developed identity‐safety in a week‐long residential creative design program that predominantly serves adolescent girls of color. We focus specifically on how participants perceive the processes involved in developing an identity‐safe community, youth engagement with this collaborative experience, and the links participants make between identity safety within a group and adolescent development. We explore these complexities through daily interviews with youth and adult participants, aged 13–28 years, over the course of the program. In the analysis, we were particularly attentive to experiences involved in developing identity‐safety and its impact on participants' understanding of themselves as program participants. Taking a cue from developmental psychology, we investigated how an inclusive community has important implications for psychological health, positive risk‐taking (Duell et al., 2018), and critical consciousness in underrepresented youth (Clonan‐Roy et al., 2016).

### 1.1 Community, identity safety, and youth development

Community is a social and psychological construct that captures the dynamic processes that create shared bonds and experiences among adolescents (Antonishak et al., 2005; Bronfenbrenner, 1979, 1995). Research suggests that supportive communities allow youth to meet their own personal and social needs: they provide a space for youth to create environments where they feel safe and valued; they build the competencies that will allow youth to have fulfilling daily lives; and they provide opportunities for youth to develop effective communication and leadership skills (Henderson et al., 2007; Lerner, 2004; Nitzberg, 2005). The social support that young people receive in these contexts has been associated with positive psychological outcomes, such as buffering the harmful effects of stressful events and encouraging the development of resilience (Chu et al., 2010; Stanton‐Salazar & Spina, 2005; Sterrett et al., 2011).

Although supportive communities benefit young people from a variety of backgrounds, underrepresented youth in particular need affirmative spaces where they can develop a sense of belonging (Clonan‐Roy et al., 2016). Adolescent girls, youth of color, and LGBTQIA+ youth often experience threats to their self‐worth and confidence due to the social invalidation of their gender, race, and sexual orientation status (e.g., Clonan‐Roy et al., 2016; Hershberger & D'Augelli, 2000; Kumagai & Lypson, 2009; Phinney, 1993). These experiences are compounded for adolescent girls with multiple marginalized identities due to interlocking structures of inequity (Clonan‐Roy et al., 2016; Crenshaw, 1989). Involvement in communities may therefore be important for the psychological health of youth who tend to receive messages that they are “less than” majority groups (Lardier et al., 2018). Indeed, participation in communities has developmental benefits—for example, research shows that for some girls of color, self‐worth and self‐esteem are connected to their sense of belonging in their communities (Pastor et al., 1996) and their sense of how their experiences are validated by others (Way, 1995, 1998).

One way that communities can build a sense of belonging is by creating identity‐safe environments. Research suggests that when young people perceive an environment as threatening rather than safe, they may expect that their social identities will evoke rejection and choose to conceal their authentic selves from others (Gamarel et al., 2014). Identity‐safe environments offset the possibility of threat by signaling that an individual “can function in [a given] setting without fear that [her] social identity will evoke devaluation and interference” (Steele et al., 2002, p. 425). Unlike other settings that may evoke a colorblind perspective, identity‐safe environments acknowledge variability in social identity and celebrate those differences (Goyer et al., 2019).

Identity‐safe environments may have positive developmental consequences for underrepresented youth, given the negative stereotypes and contextual stressors that they experience (Ely & Thomas, 2001; Woodson, 2020). Environments that promote belonging across difference allow youth to be “comfortable being [themselves]—being open, authentic, and direct—in a particular setting or role” (Edmondson, 1999, p. 491). When supportive adults and identity‐safe environments come together, underrepresented youth may be more likely to speak up and/or engage in difficult conversations because they perceive these settings as welcoming rather than threatening (Wanless, 2016). Thus, when underrepresented youth perceive contexts as supportive of different perspectives, they may explore ways of being in the world they might otherwise have kept to themselves (Ely & Thomas, 2001; Purdie‐Vaughns et al., 2008).

Identity‐safe environments articulate how contexts can promote connection in the face of the isolation that arises from alienating sociocultural stereotypes. Much of the existing empirical research on identity‐safety has been conducted within the context of schools, such as the impact of identity‐safe cues for Latina students' interest in science, technology, engineering, and math (e.g., Goyer et al., 2019; Lowe, 2020; Pietri et al., 2019). Most of this study has documented the strategies that adults use to create identity safety or youth's experiences with identity safety in the classroom (e.g., Cooper, 2013; Steele & Cohn‐Vargas, 2013), with less focus on intergenerational collaboration involved in this process. Yet intergenerational communities have been shown to have an important impact on fostering identity safety for young people (Clonan‐Roy et al., 2016; Gamarel et al., 2014). Despite these findings, little is known about how underrepresented youth develop and sustain identity‐safe communities *in collaboration with* supportive adults.

### 1.2 Conceptual framework for youth development

We draw upon Clonan‐Roy et al. (2016) theoretical model of adolescent development that centers the experiences of girls of color whose racial and ethnic groups have historically been marginalized in the United States. The model adapts the positive youth development (PYD) framework (see Lerner et al., 2005, for a review) by considering how girls of color's experiences are intimately shaped by social contexts and inextricably complicated by race, class, gender, and sexuality. The most significant adaptation to the PYD framework in this developmental model is the centering of *critical consciousness*—the ways in which girls of color develop strategies to question power structures in social environments and develop a voice to resist oppression (Asher, 2007). Similar to adolescent identity development, the growth of critical consciousness is dependent upon local communities and the presence of adult allies who encourage exploration of social identities (Clonan‐Roy et al., 2016).

An important aspect of this model is youth forming connections with older women of color who can serve as mentors (Clonan‐Roy et al., 2016). Rhodes et al. (2007) explain that, “nonparent adults and other extrafamilial sources of support have been consistently identified as contributing resilience among youth faced with a wide array of challenges.” The development of intergenerational relationships may therefore be important in providing adolescent girls of color with space to understand themselves and receive social support. We are curious if identity‐safe communities—where all participants are valued and can engage without fear of stigmatization—may also be important in the development of critical consciousness. Developing critical consciousness within a group may contribute to identity safety, while increased identity safety may make it easier for youth to engage with critical consciousness.

### 1.3 Current study

There is a growing recognition of the need for research and applied programming that contributes to our understanding of both community and identity‐safe environments (e.g., Clonan‐Roy et al., 2016; Gamarel et al., 2014). This study adds to the research literature by examining the ways in which underrepresented youth and adults *collaboratively develop* identity safety in a community‐based youth development program. We focus specifically on programmatic activities and facilitation practices that developed and sustained identity safety as well as participants' experiences with identity safety. The research questions were the following: (1) How do youth and adults engage in the process of developing an identity‐safe community? (2) How do youth and adults sustain an identity‐safe community? and (3) How does experiencing identity safety facilitate personal growth?

To answer these questions, we utilize daily interviews with youth and adult mentors who participated in a week‐long residential creative design program for adolescent girls that serves a majority of participants who are of color, from immigrant communities, from low‐income backgrounds, and/or who identify as LGBTQIA+.

## 2 METHODS

The research team conducted interviews with youth and adults about their experiences with collaboratively building community. For individuals younger than 18, the research team used an “opt‐in” parental consent procedure, such that guardians were provided with information about the study and given the option to accept their youth's participation. Youth participants under 18 provided written assent, and participants over 18 provided written consent. The research team obtained approval from an independent Institutional Review Board before recruitment.

### 2.1 Program description: Young women empowered (Y‐WE)

Y‐WE is a nonprofit youth organization that “cultivates the power of diverse young women¹ to be creative leaders and courageous changemakers through transformative programs within a collaborative community” (Young Women Empowered, n.d.). Y‐WE serves this mission by offering experiential learning opportunities through leadership classes and summer camps. In every program, Y‐WE uses an intergenerational community mentoring model, meaning there are mentors of all ages (19–75+) who are working alongside the youth through the support of staff and facilitators. Of the over 700 youth Y‐WE served in the 2018–2019 school year, 70% are first‐ or second‐generation immigrants; 85% are youth of color; and 90% are low‐income. Most staff and mentors identify as first‐ or second‐generation immigrants, LGBTQIA+, and/or racial and ethnic minorities.

The program of focus in the current research is Y‐WE Create—a week‐long residential creative design camp that empowers young women to create projects ranging from visual art to dance. Adult community members play key roles in organizing and coordinating the camp program, which serves approximately 50 youth every year. The program uses clearly defined roles for adults: (1) Mentors participate alongside the youth in activities and are most acutely aware of individual needs; (2) Social workers connect with youth one‐on‐one when their needs are greater than a mentor can meet; (3) Facilitators are responsible for creating and leading programming at camp; and (4) Camp directors oversee the programming and manage ongoing needs of youth and staff. Although adults have different roles at camp, they all serve in a group mentoring capacity to youth.

There are various ways that Y‐WE Create is developmentally and culturally tailored towards underrepresented youth. First, Y‐WE Create centers the experiences of young women who often lack access to camp and leadership programs. This means focusing specifically on whether the camp's work (e.g., programming and mentorship practices) effectively address the needs of participants who are of color, from immigrant communities, from low‐income backgrounds, and who identify as LGBTQIA+. Second, their programming links racial injustice to environmental and economic injustice, as well as patriarchy and homophobia. They interrogate pervasive assumptions of racism and sexism in their programming with the aim of creating a community space that welcomes the lived experience of all participants. Third, Y‐WE Create is predominantly led by women of color who focus on creating meaningful engagement through culturally relevant programs. Mentors and staff actively encourage youth to explore and learn from each other's backgrounds and experiences as well as speak openly about their beliefs.

### 2.2 Participants

In partnership with the Y‐WE staff, the research team selected three youth (two youth new to camp and one youth returning to camp) and two adult mentors (one mentor new to camp and one mentor returning to camp) to participate in daily interviews. Several factors informed participant selection. First, we aimed to explore the experiences of returning youth who have established social networks at camp and new youth who have little knowledge of the Y‐WE community. Second, we aimed for participants to reflect the diverse racial/ethnic identities of youth and adults at camp. After conducting intake interviews, the Y‐WE director selected youth and adults with different racial/ethnic identities (e.g., Black, Latinx, and multiracial) as potential participants for this study. Finally, we wanted to capture the experiences of adult staff with different roles at camp to gather unique perspectives about youth–adult partnerships.

The Y‐WE director provided the research team with a list of youth and adults who fit the selection criteria and who expressed interest in this study study. Potential participants (and if applicable, parents/guardians) were approached by the research team before the start of camp with information about the study. Participants received a $100 Amazon gift card as compensation for their time.

The structure of camp activities impacted the decision to interview five participants. First, the daily schedule for youth and adults included structured activities with limited breaks. To ensure that participants had a break to meet their individual needs, the research team scheduled interviews during transitions between activities, ultimately limiting the number of participants who could be interviewed. Second, we aimed to balance the number of researchers at camp with Y‐WE's goal of creating a safe space for youth. In partnership with the Y‐WE director, the research team limited the number of researchers at camp to two adult women to meet this goal. Finally, the research team interviewed the same participants daily to examine their full range of experiences. Interviewing different participants daily would have increased sample size, but we would not have been able to capture participants' arc of experiences with co‐creating community.

Interview participants came from diverse backgrounds and served different roles at camp. Saroya is a 15‐year‐old Latinx camper who is new to Y‐WE Create. Ariana is a 13‐year‐old Black and Mexican camper who is new to camp. Kayla is an 18‐year‐old multiracial camper who is returning to Y‐WE Create. Marie is a 25‐year‐old biracial/South Asian American social worker who is new to camp. Alejandra is a 28‐year‐old Latinx mentor who is returning to camp. Pseudonyms are used to protect the identities of participants, and race/ethnicity labels were self‐described by participants.

### 2.3 Procedure

Two women researchers were trained to conduct interviews. At the beginning of camp, interviewers connected with participants to introduce themselves and discuss the interview process. The researchers conducted in‐depth interviews in a private space designated solely for this purpose. Participants were informed that they could skip questions or stop the interview anytime. Each interview was approximately 20 minutes in length and happened at the same time daily (i.e., each youth took part in six interviews; each mentor took part in seven interviews). In total, the research team conducted 32 interviews over the course of camp.

The research team created a semistructured interview protocol in partnership with the program directors. The research team used questions as a guide for conducting the interviews with the understanding that flexibility is integral in interviewing, especially when asking participants to recall personal experiences (McIntosh & Morse, 2015). Interviews covered topics about community‐building, peer relationships, mentor support, and challenges at camp. Since interviews took place daily, questions were able to capture participant experiences across the program as they engaged in community building. At the beginning of every interview, participants were asked a general reflection question about their experiences (e.g., high and low points of the day). The remaining questions focused on a theme of the day, such as self‐limiting beliefs or creating community at camp. At the end of every interview, participants were asked a general reflection question about camp. See Table 1, for example interview questions.

**Table 1:** Example interview questions by day

Day	Main questions
0	What's one thing you're nervous about? What's one thing you're excited about? What's one hope you have for this week?
1	What's one thing you're nervous about? What's one thing you're excited about? What's one hope you have for this week?
2	What's a self‐limiting belief you have about yourself? How are you going to work on that this week?
3	What are you doing to co‐create a community?
4	We made a community agreement to take risks towards connection. How have you done that this week?
5	What have you personally done to help work on losing your self‐limiting beliefs?
6	Open‐ended check in about camp experience.

*Note*: On Day 0, adults and out‐of‐state youth arrive to camp. On Day 1, all youth arrive to camp. On Day 6, all adults and youth leave camp.

### 2.4 Analysis

All interviews were video‐recorded and transcribed verbatim. We used an inductive thematic analysis to explore participants' descriptions of building community at camp (Braun & Clarke, 2006). Coding and analysis followed a three‐step process. First, the authors grouped participants' responses and identified potential emergent themes. We collectively discussed the data and sorted them into more refined emergent themes. Second, we organized data within these categories into subcategories. Themes were created from subcategory codes. The third stage involved refining themes to ensure that each theme had sufficient supporting data. In the third stage, we focused on interpreting the meanings associated with each theme.

It is important to highlight that we do not report the frequency of themes found in the data. Many qualitative scholars have asserted that frequency counts of thematic codes misrepresent the methodological contributions of qualitative research (Brinkmann, 2015; Gergen et al., 2015; Valsiner, 2014). Qualitative researchers strive to gain insight into how participants articulate their lived experiences; as such, we did not want to equate frequency of a particular idea with its meaningfulness (Clarke & Braun, 2013).

## 3 RESULTS

Analyses of the interview data revealed the processes of collaboratively developing and sustaining identity safety at camp. Participants discussed developing identity safety through programmatic activities, authentic interactions between youth and adults, and open communication. Youth and adults described sustaining this environment through formal social support channels, mutual support in group settings, and peer support. These experiences set the foundation for youth and adults to engage in personal growth through risk‐taking and self‐reflection. Since developing an identity‐safe community was a collaborative process, we present youth and adult experiences together in the results. See Table 2, for a summary of themes.

**Table 2:** Summary of themes and programmatic activities

Theme	Description of theme	Example quote and programmatic activities
Developing identity safety: Cocreating community across differences	Programmatic activities that helped to establish an identity‐safe environment, mentors' facilitation strategies for developing identity safety as well as youth's perspectives with experiencing and contributing to these activities and approaches	“[Creating community] means modeling self‐acceptance, humanizing everyone throughout the day.”
		*Example activities*: Radical Welcome, I Am Posters, Y‐WE Family Groups
Sustaining identity safety: Giving and receiving social support	“Adult mentors' support strategies (i.e., formal support settings, mutual support in group settings, mediation in peer conflict) as well as youth's role in building social support channels with peers (i.e., peer support, informal roles).”	“It was amazing that people were able to hold space for other people, the youth for themselves and for their peers.”
		*Example Activities*: Self‐Care Sanctuary, Heart Circle, Small Group Activities with Peers
Effects of identity safety: Balancing discomfort with personal growth	Internal positive risks (i.e., overcoming imposter syndrome and self‐limiting beliefs, communication patterns) and external positive risks (i.e., attending camp, playing a musical instrument).	“I think the strongest one [self‐limiting belief] in my life for the last few years has been self‐hatred and body shame … I'm actually addressing that in a workshop at Y‐WE Create.”

### 3.1 Developing identity safety: Cocreating community across differences

Mentors and youth discussed the experience of collaboratively developing a community that elicited a sense of belonging and inclusion. This theme includes descriptions of programmatic activities that helped to establish an identity‐safe environment, mentors' facilitation strategies for developing identity safety as well as youth's perspectives with experiencing and contributing to these activities and approaches.

Participants described the importance of creating a community where youth and adults felt comfortable being themselves as an integral aspect of identity‐safety. The process of developing identity safety began on the first day when mentors introduced youth to the concept of *Radical Welcome*—a collaborative experience that celebrated the unique voices and identities of youth and adults. Radical Welcome invited participants into the process of co‐creating community through three activities: (1) an invocation that acknowledged the diversity of language, gender, sexual orientation, race/ethnicity, social class, and ability (see supplement); (2) rapport‐building activities (e.g., sharing personal stories in small groups); and (3) the collaborative development of community guidelines. Marie (28‐year‐old multiracial social worker and mentor) talked about the importance of Radical Welcome so that youth and adults experienced safety to express their whole selves at camp:

I think Adrienne [camp facilitator] did a really good job at tone setting and being really welcoming and making sure everyone was connecting, not necessarily groups of people who already knew each other from past experiences or people who know each other from their personal lives. I feel like we all got a chance to know each other through being in circle, sharing personal stories, and some of those getting to know you through introductory exercises.

Echoing Marie's observations about the first day of camp, youth expressed that tone setting helped them overcome anxieties about being accepted in their new environment. For example, Arianna (13‐year‐old Black and Mexican youth) discussed experiencing anxiety about peer acceptance on the first day of camp and the impact of tone setting on bolstering her sense of belonging: “I like how Adrienne is telling everyone that they should accept themselves and not feel ashamed about themselves. Like that felt good and I'm just like 'hey, I can actually be myself here.'” As Kayla (18‐year‐old multiracial youth) described, the centering of youth experience during the first day of camp was also integral in her self‐expression: “It's been helpful to see youth stepping up, because that just creates space for others to step up. When someone I have seen not talking much speaks out, we all feel a little more comfortable … I can talk to anyone because of the trust that Adrienne and Tonya [camp directors] set up for us on the first day.”

Another way that participants developed identity safety was through challenging traditional social structures that privilege the experiences of certain groups of people. Developing the skills to critically analyze power relationships in the social world, and the ways in which interactional forms of gendered and racial/ethnic oppression shapes lives, can empower adolescent girls of color to speak about their own experiences (Clonan‐Roy et al., 2016; Ward, 2007). An example of sharing life stories was the *I Am Posters*, an activity that took place on the second day of camp intended for youth to introduce themselves. Although mentors hoped that the activity would elicit positive affirmations, youth expressed self‐doubt about their bodies given the pressures of (White, heterosexual) Western beauty standards. To redirect youth's self‐doubt, Marie explained that Adrienne educated youth on cultural values that may impact their attitudes:

Adrienne called out and named how most of us would immediately target the negative things that we saw in the photo and educated everyone about negativity bias, how normal that is, not turning the conversation about how you should love yourself or you should point out positive things but more this is really valid. Our brains are literally wired to identify negative attributes or stressful things in our environment. That was really good to name for some youth because I immediately heard understanding around the room.

Adrienne invited the adults to share their posters which featured photos of their adolescent selves and advice they wished they could give their younger selves. Youth recounted that mentors' stories helped them identify commonalities with their own experiences. Ultimately, participants described that conversation around these topics helped establish identity safety because they could openly share expressions of frustration and sadness felt in response to the external world.

Facilitated activities with mentors helped some youth re‐evaluate norms about friendships and in turn, encouraged them to respect the unique lived experiences of peers. Participants described small group activities such as *WE groups*—predetermined groups of youth and mentors who met daily to engage in group discussions and games—as important in reframing their approaches to peer acceptance. For example, Kayla talked about the process of unlearning norms about cliques during small group activities:

I think when a lot of people come here, when they hear summer camp or you know, people my age interacting, it's kind of a, almost like a school vibe and like, ‘Okay, we've got to form cliques. We've got to speak in this way and interact in this way that I don't think they think is forced, but it is a societal norm that they don't even know they're following. And Y‐WE is not that, you know, I think it's really sometimes confusing and disrupting for them when it's like, ‘we're not actually going to blame you for these things and we're not going to kick you out if you are disruptive, but we need you to work as a group, and work for the group and yourself.'

An important aspect of Kayla's experience is that bonding activities at camp (e.g., creative play with other youth, small and large peer discussions around a specific topic) challenged her to develop a sense of responsibility across the whole group, not just for the people to whom she naturally gravitated. Widening youth's friendship networks was an important aspect in developing a community where everyone felt respected and valued. As Ariana reflected, bonding activities can also impact how youth perceive themselves: “[WE groups] is kind of like talking to your family, except it's with other people that aren't your family … a lot of people at my school are really fake and stuff like that. And these people here, they're real. And I think that's really cool because there's people just like me here.”

Beyond programmatic activities, interviews revealed that different life perspectives brought by people from across the age spectrum facilitated close bonds and in turn, helped establish safety in the community. For mentors, this process often meant practicing authenticity during their interactions with youth by discussing their identities, backgrounds, and life histories. Such relationships with adult women can provide adolescent girls of color with guidance and support as they become aware of the differences in power present in social relationships (Clonan‐Roy et al., 2016). Alejandra (28‐year‐old Latinx mentor) talked about the importance of authenticity in youth‐adult partnerships to build safety in the community:

I want them to learn these things that I wish I would've known at their age but I don't think just saying it gets the message through. I think *doing* is what gets the message through. So maybe me, myself being more vulnerable, being more open, being more, again my authentic self and I am hoping that reflects or it shows the other youth and it just inspires them to be like, ‘well if this older person who…' Because when I was a kid, I thought the adults had it together. If this older person admits to making mistakes and to not really feel like they got it together all the time and to having ups and downs then it's okay, like I could do that stuff too.

Alejandra reflected on the role of mentors in supporting youth to reflect on their identities and experiences in their social environments. As Marie highlighted, humanizing experiences of mentors can ultimately help youth feel respected in the camp community: “[We need to] model self‐acceptance, humanize everyone throughout the day … I think taking questions seriously is one way to humanize people so that they don't feel left out or belittled or dismissed. Which, as a young person, you typically feel around adults.”

Finally, youth recounted that open communication was important in developing safety at camp to address trauma, adversity, and other challenges that youth encounter. Youth consistently described mentors as modeling effective communication strategies during daily activities, and youth incorporated these strategies in their own approaches to community building. For youth who experienced anxiety about attending camp, open communication between youth and adults helped alleviate fears of social rejection. Saroya (15‐year‐old Latinx youth) characterized the community that youth and mentors built together as instrumental in “opening [her]self up and actually showing [her] real self to others”:

A lot of people tell a story that they are going to be judged a lot, so that's kind of like my story too. I feel like when I first talk to a person, they're going to judge me, so I don't usually talk to new people. I feel like the first thing is communication. We have to communicate with each other, respect each other, so we can be a good community.

Saroya's experience exemplifies the ways in which the camp community strengthened participation, and in turn, helped youth re‐evaluate negative beliefs about themselves. Participants recognized over the course of the week that community was not just about creating relationships; it was also about building a safe space, both physical and emotional, to better communicate empathy to themselves and others. As Marie expressed, the sense of acceptance helped some youth “articulate stories and narratives that have held [them] back. And I hear youth speaking in ways that are less self‐critical … it matters that they're spending hours every day being challenged to unlearn some stuff.” Ultimately, the development of identity safety encouraged youth to speak in an authentic voice because they were given a supportive space to fully explore their values and identities (Belenky et al., 1986; Clonan‐Roy et al., 2016).

### 3.2 Sustaining identity safety: giving and receiving social support

Participants described sustaining identity safety through giving and receiving social support. We define social support as the social resources and social networks that participants used for advice, approval, or assistance; it includes information that one is cared for and valued as part of the community (Cicognani, 2011). This theme includes descriptions of adult mentors' support strategies (i.e., formal support structures, mutual support in group settings, mediation in peer conflict) as well as youth's role in building social support channels with peers (i.e., peer support, informal roles).

Throughout the interviews, mentors and youth discussed sustaining an identity‐safe community through the development of formal and informal social support channels. One way that participants experienced social support was through the *self‐care sanctuary*—a quiet space where youth could focus on themselves through reflection, art projects, or talking with a social worker. For youth who experienced anxiety or tension at camp, the self‐care sanctuary provided a space to talk about their experiences without fear of judgment from peers and mentors (e.g., disbelief of experiences, minimizing emotions). Marie reflected on the importance of the self‐care sanctuary in sustaining a culture of mutual appreciation and individual importance:

I think I had one‐on‐ones with four youth yesterday. We had anxiety, some symptoms of panic attacks coming up for certain campers, and migraines. I see them using the self‐care sanctuary as a place to sit with things and it's the least structured space in our camp. I think they really crave that at times, just a space to go sit with a coloring book for an hour. I see them using it and asking ways to modify it.

Youth described the self‐care sanctuary as providing a communal space to interrogate self‐limiting beliefs with others. Often, it took mentors and peers being vulnerable in front of others for participants to feel safe and share their own experiences. Youth described these moments as reciprocal in that vulnerability helped sustain an environment where peers felt safe to engage in similar behaviors. For example, Kayla described the self‐care sanctuary as providing a safe space to reflect on her body insecurities and receive social support from others: “Because you're really in a safe space completely. Going into the sanctuary, I just felt so completely relaxed and in my body. I didn't have to adjust, at all, what I was feeling or looking like, because I felt so safe and that's just the feeling of the whole camp.”

The development of identity‐safety within the community encouraged participants to provide mutual support to one another in larger group settings. Participants described mutual support as being there for one another by (1) providing encouragement and guidance; (2) listening to other people's life experiences; and (3) offering emotional support in moments of vulnerability. A key activity where participants engaged in mutual support was the *Heart Circle* that took place after the midpoint of camp—a collaborative experience with the entire community that invited youth to share personal stories (e.g., barriers, struggles, joys, and disappointments) in a space that was loving and affirming. Alejandra discussed the “domino effect” of this activity for youth to share their experiences collectively as well as receive support from others:

The second youth's story felt like a gateway. And then I felt like it kept coming and coming and coming and coming. For a second, you could hear everyone sobbing in the room. I think it was really powerful. It was amazing that people were able to hold that space for other people, the youth for themselves and for their peers. I looked around and mentors were teary‐eyed as well because stuff like that's hard to hear.

The sense of belonging established throughout the week communicated to youth that it was safe to share their experiences with the community and provide encouragement to their peers. As Ariana reflected, the mutual support that youth and mentors collaboratively developed was instrumental in maintaining safety at camp: “[The Heart Circle] was just making space for individuals to be fully seen … I think that it's due a lot in part to the space we've made, and making that space feel more welcoming and more comfortable.”

Although participants reported several positive experiences, some youth confronted conflict with their peers about belonging at camp. During moments of conflict, mentors' emotional support and mediation techniques helped youth resolve disagreements and re‐establish a sense of safety. For example, Marie described a situation where Crystal (17‐year‐old white youth) and Maya (13‐year‐old Black youth) experienced conflict about group inclusion. Crystal reported feeling excluded from social activities with Maya, while Maya felt that she included Crystal in her friend group. While mentors provided emotional support to Crystal and Maya, they ultimately encouraged the youth to work collectively towards resolution:

Adrienne explained that we're in the storming phase and that the group will develop its own immune response to its own sickness … It's super valid to want to jump in as the adult or person in charge in certain situations and attempt to fix or resolve things. But youth are resourceful and learning through conflict and it could transform without us intentionally trying to fix or solve or do anything about it.

Both youths eventually resolved their disagreement through talking with each other and spending the afternoon together. Ultimately, by not directly intervening in peer conflict, mentors encouraged youth to develop a sense of responsibility with one another.

While mentors played an important role in fostering social support, youth also actively contributed to the community as a way to cultivate belonging. Some youth expressed that peer social support was integral in bolstering their own feelings of acceptance at camp. For example, Ariana talked about the reciprocal relationship between receiving social support and feeling free to express herself at camp: “I love Joey [camper], they are saying I'm beautiful and I'm awesome‐they're always‐ checking up on me… It's like at school I have to be a lot quieter because kids can be really mean. But here I can just be myself. I can be my own human. My spirit can just run free here.”

Youth also highlighted the importance of smaller moments—such as taking a walk or bonding over shared interests with peers—in sustaining a safe environment at camp. For youth who were new to Y‐WE, these experiences helped them feel accepted in the community. For example, Saroya was new to camp and only knew a few other young people. She recounted what this experience looked like for her:

I made a new friend…We learned a little bit about each other and then we became friends. It was really nice. Me and my other friend were talking about it and we're just like “oh there's really nice people here. They're so outgoing” And we're just like wow. But sometimes, we also still feel like we are going to be judged, so we're just getting there little by little.

For Soraya, building friendships helped her slowly overcome fear of judgment by her peers. Because girls are often socialized to be “steeped in a culture that wants to keep women at each other's throats” (Kayla), it may take time for new youth to develop trust with peers and feel safety to share vulnerable feelings with the camp community. Ultimately, the development of peer relationships helped some youth explore shared and different histories with others.

Finally, participants recognized that to sustain generative communities where people know they belong and are cared for requires intention. One way that participants intentionally sustained safety was through embodying informal roles at camp. Participants described these roles as self‐directed and emerging from a desire to bolster a culture of acceptance among mentors and youth. For example, Alejandra hoped to cultivate a welcoming space for youth: “I almost just want them to feel like they've come home … Like they're welcome and everyone's really happy to see them.” Ariana described her role as bringing joy to the community: “My role here is to make people smile and feel like they're worth a lot. To make them feel like they have a purpose on this earth.” Kayla reflected on her role as a peer mentor for younger campers: “I'm someone a youth can turn to and ask questions and work things out with. Because I remember it was hard for me sometimes to reach out to adults occasionally just because I wasn't used to it when I first came to Y‐WE.” Taken together, these informal roles offered participants an avenue to take responsibility in validating the experiences and backgrounds of others.

### 3.3 Effects of identity safety: balancing discomfort with personal growth

Given the development of inclusion and trust in the community, participants described feeling safe to engage in personal growth opportunities at camp. A common thread throughout participant personal growth descriptions was engaging in *positive risk‐taking*—socially acceptable and constructive risks such as overcoming a fear, enrolling in a challenging course, or starting a new friendship (Duell et al., 2018). Participants described positive risk‐taking as looking beyond the potential physical effects of risk, such as failing at an activity or getting lost, to consider the mental aspects of risk, such as the effects on wellbeing or self‐identity. This theme includes descriptions of internal risks (i.e., overcoming imposter syndrome and self‐limiting beliefs, communication patterns) and external risks (i.e., attending camp, playing a musical instrument).

One way that participants engaged in personal growth was through taking a risk to overcome feelings of inadequacy. A commonality in participant descriptions was that they often experienced doubts about their skills, accomplishments, or talents at the start of camp. For example, Marie expressed that she initially struggled with imposter syndrome because she questioned her qualifications as a social worker. However, a sense of safety and belonging developed over the course of the week that helped her overcome this fear:

I generally struggle with imposter syndrome. And I'm not always my own ally in moments where I feel unprepared or I feel like I'm not fully qualified or the right person to be working with youth, doing mental health work … But I think that there's something to be said for sitting with your hard emotions and self‐narratives as they come up, being radically accepting and almost just waiting them out. And I feel like something shifted and I feel more comfortable being here. Way more confident, way more ready to step into whatever role that I might be called into.

Although this experience initially elicited discomfort, Marie took a positive risk by engaging in critical self‐reflection as a way to confront anxieties and achieve personal growth in her role as a social worker. Alejandra echoed Marie's experiences with safety as foundational in examining her strengths and abilities as a mentor: “Sometimes I don't give myself enough credit for working with kids. I'm just like, oh, anybody could do it … Taking those mirrors [at camp] and really absorbing what those mirrors say, I belong here.”

Youth took positive risks within the community through interrogating self‐limiting beliefs about themselves. Youth experiences ranged from reframing negative thoughts about their bodies to overcoming fears about being away from home. For example, after the *I Am Posters* activity, Kayla expressed that body shame had a powerful influence on how she felt emotionally and physically. Toward the end of camp, she decided to lead a body image workshop so she could engage in conversation with other youth. Since she experienced safety at camp, she hoped to bolster her confidence through taking a risk to collectively discuss body image concerns. Her experience exemplifies how increased identity safety may make it easier for youth to engage with critical self‐reflection:

One of my issues is that when I have a negative thought or something holds me back, I kind of have this other thought that says there's no use fighting it. It's going to happen anyway. And I think that's a really limiting belief for me is it's not worth it to fight back. I think the strongest one in my life for the last few years has been self‐hatred and body shame, and that's just been a constant in my life and I'm actually addressing that in a workshop coming up at camp.

Similar to Kayla, Saroya demonstrated that personal growth can involve the ability to take notice of thoughts, to step back and view the bigger picture, and to decide how to act based on that more realistic perspective. Saroya described her trip from Las Vegas to the Pacific Northwest as challenging due to discomfort about her first time being away from home. She was able to confront these fears through reflecting on her accomplishments with youth and mentors, such as flying for the first time and taking a ferry to camp: “Looking at the logistics of it [coming from Las Vegas to camp] makes me think, 'oh I can do that.' … Yeah I have a feeling I can do more now, because I did all of that.” Taken together, both Saroya and Kayla's risk‐taking show that a sense of safety nurtured at camp can lead to positive action, which, in turn, may bolster confidence and enhance self‐esteem.

Another way that participants engaged in personal growth was through challenging cultural norms around language. Participants often talked about the process of unlearning socialization practices—such as using competitive language or silencing their unique experiences—as having a positive effect on their wellbeing as women, LGBTQIA + youth, and/or a person of color. These experiences can be seen as risks because they required participants to reflect on internalized thought processes and make an effort to change them. For example, Marie recounted that the camp community helped her re‐evaluate her communication patterns and as a result, helped her relate to others across difference:

I'm getting a lot of practice being careful, not censoring myself, and speaking more gently. The way I've experienced being socialized and how I communicate is very competitive and comparative and conflicting in terms of how I relate to other people. I haven't been taught to relate across difference. That's something I've had to learn [at camp] …I'm learning to not lean into my perfectionistic side that really beats myself up every time that I've made a mistake.

Interactions with youth and mentors motivated Marie to develop a new way of using language to express herself. Ariana also described language as important in creating an atmosphere where young people felt allowed to take risks. When other youth shared personal experiences with their peers, she felt empowered to also take a risk and contribute to these conversations: “A lot of people didn't talk as much in the beginning [of camp]. Then people started coming forward and saying the fears they were scared of for this week. They started to loosen up and encourage me to actually step forward.”

Finally, youth engaged in personal growth at camp by taking a risk to conquer a long‐held fear. These experiences ranged from performing at the talent show to overcoming anxieties about spiders. For example, Ariana talked in‐depth over the course of the week about how she wanted to play her clarinet but often experienced anxiety while performing. She expressed a willingness to rehearse daily in the hope that she would perform for her peers:

It [playing my clarinet] is scary to think about, because I'm going to be in front of everybody and sometimes I squeak or I get the note wrong … I really, really, really have to rehearse today … I don't like being in front of people because I can be very awkward and sometimes I might stare a lot … I'm hoping to leave some of my stage fright behind because I'm going to be playing in front of people and that's a big thing for me. Like a really huge thing.

Although Ariana experienced anxiety about participating in the talent show, she expressed a willingness to address and revise negative perceptions related to performing. As she grew more comfortable being herself at camp, she eventually played her clarinet at the talent show despite the risk of failure. Importantly, this experience had a positive impact on her self‐identity beyond camp: “I'm happy because this is really gonna change me. I feel a lot better being in front of people a tiny bit, and I think I'll teach people other ways of art.”

## 4 DISCUSSION

The aim of this study was to examine the ways in which youth and adults developed and sustained an identity‐safe community in a youth development program. We add to the literature by understanding the strategies involved in creating an identity‐safe community as well as youth and adult engagement with this experience. This study revealed that participants developed an identity‐safe community through programmatic activities, engaging in authentic interactions, and practicing open communication. Participants described sustaining identity safety through formal social support channels, mutual support in group settings, and peer support. These experiences set the foundation for youth and adults to engage in personal growth through positive risk‐taking and self‐reflection. Below, we consider the importance of identity‐safe communities and intergenerational bonds for adolescent development; the connections between identity safety and positive risk‐taking; and the implications for scholars and practitioners.

### 4.1 Girls of color, identity safety, and adolescent development

Adolescence is the developmental period in which young people establish an identity by exploring who they are, how they fit into their communities, and who they will become in the future (Erikson, 1968). This exploration process tends to be more complex for adolescent girls, racial and ethnic minorities, and LGBTQIA + youth due to their social location (e.g., Hershberger & D'Augelli, 2000; Phinney, 1993). Research indicates that adolescent girls of color—whose experiences are shaped by their social contexts and complicated by race, class, gender, and sexuality—may need to develop additional strategies as a way to resist multiple oppressions that youth of color experience (Clonan‐Roy et al., 2016; Ward, 2007).

In their adaptation of the PYD framework (see Lerner et al., 2005, for a review), Clonan‐Roy et al. (2016) argued that the development of critical consciousness is fundamental for adolescent girls of color to fully recognize their potential. The authors explain that critical consciousness is the ability to question power structures in social environments, and more subtle forms of gendered and racialized marginalization. The development of critical consciousness is ultimately dependent upon context: one's environment, personal experiences, and the presence (or absence) of supportive adult allies. The core of this developmental model focuses not only on how adolescent girls of color develop the capacity to critically question inequities in the social world, but also their use of strategies to resist oppression and work towards personal growth (Asher, 2007).

Our results suggest that increased identity safety may make it easier for youth to engage with critical consciousness, while developing critical consciousness within a group may contribute to identity safety. Participants described programmatic activities and authentic youth–adult partnerships as signaling safety across difference, which allowed them to fully explore their experiences and (often marginalized) positions in their social environments. When youth engaged in activities that developed critical consciousness, they described a sense of validation and safety from sharing these experiences with peers. The interdependence between identity safety and critical consciousness indicate that communities can serve as what Nakkula and Toshalis (2006) call *homespaces*—a space where adolescent girls of color can have “safe exchanges of ideas, intimate discussions of desire, and expressions of anger and frustration felt in response to the external world” (p. 107). These types of contexts offer youth the opportunity to develop critical consciousness and experience identity safety by interrogating larger sociocultural messages about different social identities (e.g., race and gender; Pastor et al., 1996) and growing their sense of feeling validated by others (Way,^1995^, ^1998^).

Our results offer insights for the construction of identity‐safe communities as environments in which intergenerational mentors can empower adolescent girls of color to develop a reflexive awareness of their social environments—an important factor in the development of critical consciousness. A common thread throughout the interviews was that mentors described modeling acceptance, open communication, and sharing personal experiences during their interactions with youth. For many of the young people, supportive relationships with mentors of color influenced their ability to speak authentically (Brown & Gilligan, 1992) and view their experiences with a critical eye. When adolescents sense that adults respect and share their lived experiences, they may internalize this positive appraisal and increase their own feelings of self‐worth (Rhodes et al., 2007). Since girls of color live in a society in which both women and people of color often are marginalized, supportive relationships with mentors of color may provide opportunities for youth to explore their self‐worth and confidence in a safe environment (Banister & Leadbeater, 2007).

In addition to intergenerational mentorship, we found that the collaborative experience of creating community emerged as crucial for participants to acknowledge their experiences as legitimate and valuable. Adults encouraged youth to actively take part in building community through facilitation strategies, such as establishing an inclusive environment (e.g., tone setting), planning opportunities for connection (e.g., structured activities), and cultivating engagement opportunities for youth to take positive risks (e.g., structured group conversations). Since adults participated *alongside* youth in all activities, youth described feeling empowered to build and sustain identity‐safety through establishing mutual rules of respect, ways of engaging with others, and informal roles of social support. Collaboratively establishing identity‐safety impacted youth experiences in important ways: youth described wanting to speak up and/or engage in difficult conversations because they perceived the community as welcoming rather than threatening (Ely & Thomas, 2001; Wanless, 2016); and youth described seeking out social support that nurtured their existing strengths and allowed them to challenge self‐limiting beliefs about themselves. Overall, collaboratively establishing identity safety helped empower participants to share their experiences and develop an awareness of their social worlds.

### 4.2 Identity safety, psychological safety, and positive risk‐taking

Identity‐safe communities have the potential to provide a foundation where individuals can build skills and expand their understanding of themselves (Bialeschki et al., 2007). One way that individuals engaged in this personal growth process was through positive risk‐taking (Duell et al., 2018). In the current research, participants described positive risk‐taking as looking beyond the potential physical effects of risk (e.g., playing a new game) to consider the mental aspects of risk (e.g., engaging in a challenging conversation). The experience of positive risk‐taking was different depending on the individual needs of youth and adults; however, a thread throughout these experiences was a willingness for participants to try new things they might not like or at which they may fail.

A willingness to engage in positive risk‐taking aligns with the literature on psychological safety. Edmondson and Lei (2014) define psychological safety as the extent to which people feel comfortable sharing different perspectives, despite the possibility of discomfort or harm. Stated differently, it is not necessarily the absence of risk that makes a setting psychologically safe, but rather the opportunity for participants to engage in risks that encourage personal growth (Williams et al., 2016). Our findings underscore that as participants integrated into a community supportive of their lived experiences, they expressed a willingness to take positive risks ranging from overcoming feelings of inadequacy (e.g., imposter syndrome) to developing a sense of self (e.g., confidence). These results suggest that as individuals and identity‐safe environments come together to create a sense of psychological safety, youth and adults may be more free to engage in self‐exploration, without fear of rejection (Wanless, 2016).

An important insight in this study is the connection between identity‐safe environments and psychological safety for youth and adults. Research in the fields of business and organizational leadership have found that when individuals feel psychologically safe, they anticipate that positive risk‐taking will not pose a threat to their sense of self (Frazier et al., 2017). What remains unclear in the literature are the conditions that create psychological safety for youth who often experience threat due to the invalidation of their identities. Findings suggest that the creation of an identity‐safe community that validates youth's identities may be antecedents to psychological safety in applied community settings. This stands in contrast to a colorblind perspective that erases disparities related to race and other social identities when working with youth (Brown et al., 2003; Markus et al., 2000). Thus, creating communities that celebrate the complexity of experience may be important for the development of psychological safety and in turn, underrepresented youth's positive risk‐taking behaviors.

### 4.3 Limitations and implications

The findings of the present study should be interpreted in light of some important limitations. We opted for daily interviews to gather information about participant experiences over the course of the program; however, this design also resulted in a small sample size. It is possible that this approach may have underrepresented experiences of identity safety and positive risk‐taking found within the larger camp community. Similarly, the small sample size and interview questions did not allow us to examine changes over time in the development of identity safety. Future work could be structured in a way that facilitates comparisons over time in how youth think about identity safety.

While many of the activities and discussions did ask participants to reflect on their salient social identities, much of the work conducted to build and sustain an identity‐safe community in this study could be described as relational and interactional, rather than dealing directly with stable and pre‐existing (i.e., macrosocial) identities (e.g., race and ethnicity, gender identity; see Tracy & Robles, 2013). This could be an artifact of interview questions that delved into building community and friendship instead of focusing exclusively on social identities. Still, this current research is in line with work on psychological development and well‐being that asserts that fostering relational connections empowers individuals, increases sense of self‐worth and validation, and encourages knowledge of self and others (Gamarel et al., 2014; Liang & Grossman, 2007; Rhodes et al., 2007; Spencer, 2006). Future research should address the intersection between macrolevel identities and relational experiences in youth's engagement with community building and intergenerational bonds.

The study findings have unique implications for practitioners working with diverse youth. First, identity‐safe communities have the potential to encourage girls of color to interrogate different histories that impact one's experiences, an important component in the development of critical consciousness. Practitioners may want to incorporate programmatic activities that seek to understand youth's identities, experiences, and the roles they enact in their social environments. Second, the intergenerational mentoring model worked to challenge power dynamics inherent in youth–adult partnerships by having mentors participate in activities alongside youth. By capitalizing on differing life perspectives across the age spectrum, youth were able to explore their own values with mentors and build their sense of self‐worth. Third, mentors and facilitators actively challenged sociocultural expectations during group activities as a way to establish new norms at camp. This study helped create personal accountability in how youth and adults engaged with others. Finally, mentors recognized that some youth may need additional support through one‐on‐one meetings and the self‐care sanctuary. Acknowledging individual needs may help youth to meet the challenges of adolescence by providing supports that contribute to their personal growth. We believe that considering these areas in programming—nurturing the development of critical consciousness, challenging power dynamics in youth–adult partnerships, addressing sociocultural expectations, and tailored support—can help build safe communities for traditionally underserved youth.

## 5 CONCLUSION

The aim of this study was to explore the ways in which a diverse group of youth and adults collaboratively built an identity‐safe community in a week‐long residential creative design program. We found that youth and adults developed identity‐safety by practicing authenticity in daily interactions, engaging in programmatic activities that sought to understand youth's identities, and facilitating dynamic communication across intergenerational friendships. As the community developed, youth and adults engaged in providing reciprocal support to sustain safety and engaged in personal growth opportunities through taking positive risks. These collaborative processes helped establish a community of belonging, ultimately encouraging youth and adults to develop feelings of trust and connection.

### PEER REVIEW

The peer review history for this article is available at https://publons.com/publon/10.1002/jcop.22526

## Supporting information

Supporting information.

## Data Availability

Due to the nature of this study, participants of this study did not agree for their data to be shared publicly, so supporting data is not available. Many of the participants were minors at the time of data collection; given that the data is qualitative and tied to a specific youth development program, participants and their guardians did not want the full transcripts of interviews to be released to the public.

## ENDNOTE

Y‐WE is open to those who identify as women or girls or who were assigned female at birth.
