# Supplementary material for: “Here I can just be myself”: How youth and adults collaboratively develop an identity‐safe community across difference
Source: J Community Psychol. 2021 Feb 24;49(5):1024–43. doi: 10.1002/jcop.22526 (PMC8359412; doi:10.1002/jcop.22526)
Supplement: Supplementary file 1 — Supporting information. [file JCOP-49-1024-s001.docx]

**Supplement. Radical Welcome Invocation**

Welcome to Y-WE Create. More than a program, this is a rite of passage during which you will co-create a special community and discern who you are as leaders and changemakers in our community and in the world. As we enter into this collective we want to invite you to show up in the fullness of who you are.

We welcome People Indigenous to this land and people of African descent. We welcome our Latinx, Asian and Pacific Islanders, Middle Eastern, and European descendent peoples. We welcome those of mixed and multiple heritages.

We welcome all the languages spoken here: Spanish, Amharic, Vietnamese, English, Arabic, Hebrew, Japanese, Russian, and others.

We welcome Women, Transgender, Cisgender, A gender, Gender Queer People, People of all genders, folks of no gender ......

We welcome gay, lesbian, heterosexual, bisexual, pansexuals, queers or others for whom none of these labels fit.

We welcome those who come from poverty and working class, middle class, and those with owning class privilege.

We welcome those living with chronic medical conditions, visible or invisible. We welcome your abilities and your disabilities.

We welcome your bodies, your minds, your full, complicated and complex selves.

We welcome the full spectrum of your emotions. We welcome your joy, your grief, rage, indignation, curiosity, contentment, disappointment, confusion, and gratitude.

We welcome people who have never done this kind of welcome circle before and those who participate in circles like this regularly.

We welcome people who identify as religious, as atheist, as spiritual, as agnostic, as something in between or unnamed

We welcome your muses, creative inspirations, risk taking selves. We welcome our ancestors and the beautiful lineages that have created us. We welcome those who supported you to be here now.

Every part of you is welcome here, From cells to bones to spirit, From everyday challenges to ancient wisdom, Your stories, beliefs, hopes, dreams, and fears, Your community, your solitude, and your sacred relationship with yourself.

And finally we'd like to welcome the ancestors who lived in this land where most of us live. We welcome the Duwamish, the Coast Salish peoples. All of who you are is necessary and sacred and enough and you are welcome in this beloved community.
